# Supplementary material for: Identifying plant genes shaping microbiota composition in the barley rhizosphere
Source: Nat Commun. 2022 Jun 16;13:3443. doi: 10.1038/s41467-022-31022-y (PMC9203816; doi:10.1038/s41467-022-31022-y)
Supplement: Supplementary file 1 — Supplementary Information [file 41467_2022_31022_MOESM1_ESM.pdf]

Identifying plant genes shaping the microbiota composition in the barley rhizosphere

Escudero-Martinez, Coulter, *et al*.,

Supplementary Table 1. Genome-wide LOD threshold score calculated per individual taxa at ASV, genus and family level.

ASV (alpha=0.2)								
Sphingomonas env.OPS 17	*Allo.Neo.Para. Rhizobium*	*Pedobacter1*	*Paenibacillus1*	*Niastella*	*Paenibacillus2*	*Stenotrophomonas*	*Paenibacillus3*	*Streptomyces2*
6.92	8.92	8.8	3.19	6.18	3.31	15.0	3.68	3.93
Lysobacter1	*Asticcacaulis*	*Variovorax*	*Paenibacillus4*	*Bdellovibrio*	*Duganella*	*Holophaga*	*Vicinamibacterales*	*Sphingobacteri*
3.80	10.0	6.01	5.73	8.74	7.07	12.2	3.43	3.63
Lysobacter2	*Sorangium*	*Sphingopyxis*	*Chryseolinea*	*Tahibacter*	*Pedobacter2*	*Comamonadaceae*	*Chryseobacterium*	*Streptomyces3*
5.20	13.9	7.75	9.61	9.79	6.32	7.26	4.96	5.47
Pedobacter3	*Asticcacaulis1*	*Pseudomonas*	*Pseudomonas1*	*Streptomyces1*	*Lysobacter3*	*Sphingomonas*	*Nocardia*	
11.4	7.81	5.70	6.68	5.19	11.1	5.36	3.78	
**Genus (alpha=0.2)**								
Pedobacter	*Paenibacillus*	*Ramlibacter*	*Rhodanobacter*	*Asticcacaulis*	*Thermomonas*	*Variovorax*	*Dokdonella*	*Chryseobacterium*
6.61	3.09	5.20	15.5	9.83	8.79	10.5	6.65	4.34
Bdellovibrio	*Microbacterium*	*P3OB.42*	*Holophaga*	*Sorangium*	*Chryseolinea*	*Tahibacter*	*Comamonadaceae*	*Streptomyces*
4.38	4.29	12.2	11.5	12.6	7.26	8.52	4.06	3.58
**Family (alpha=0.2)**								
Sphingobacteriaceae	*Paenibacillaceae*	*Microscillaceae*	*Opitutaceae*	*Caulobacteraceae*	*Comamonadaceae*	*Chitinophagaceae*	*Alcaligenaceae*	**Nocardiaceae**
5.90	3.38	6.87	4.10	5.47	9.90	3.78	7.11	4.02
Micromonosporaceae	*Holophagaceae*	*Labraceae*	*Polyangiaceae*	*Rhodanobacteraceae*	*Weeksellaceae*	*Streptomycetaceae*	*Solirubrobacteraceae*	
3.74	13.3	8.65	12.4	11.6	3.80	3.60	4.37	

Values calculated with 1,000 permutations. Source data are provided as a Source Data file.

Supplementary Table 2. Genome-wide LOD threshold score calculated per individual taxa at ASV, genus and family level.

**ASV (alpha=0.05)**								
Sphingomonas env.OPS 17	***Allo.Neo.Para. Rhizobium***	***Pedobacter1***	***Paenibacillus1***	***Niastella***	***Paenibacillus2***	***Stenotrophomonas***	***Paenibacillus3***	***Streptomyces2***
8.37	9.15	10.3	3.91	7	4.2	17.1	4.53	4.96
Lysobacter1	***Asticcacaulis***	***Variovorax***	***Paenibacillus4***	***Bdellovibrio***	***Duganella***	***Holophaga***	***Vicinamibacterales***	***Sphingobacteriaceae***
4.78	11.3	8.01	12.8	9.29	8.70	12.2	4.23	4.79
Lysobacter2	***Sorangium***	***Sphingopyxis***	***Chryseolinea***	***Tahibacter***	***Pedobacter2***	***Comamonadaceae***	***Chryseobacterium***	***Streptomyces3***
6.63	15.4	8.80	10.5	11.54	7.97	7.97	7.19	7.43
Pedobacter3	***Asticcacaulis1***	***Pseudomonas***	***Pseudomonas1***	***Streptomyces1***	***Lysobacter3***	***Sphingomonas***	***Nocardia***	***Streptomyces4***
12.1	9.80	7.20	8.63	6.25	11.6	7.39	4.71	11.7
**Genus (alpha=0.05)**								
Pedobacter	***Paenibacillus***	***Ramlibacter***	***Rhodanobacter***	***Asticcacaulis***	***Thermomonas***	***Variovorax***	***Dokdonella***	***Chryseobacterium***
7.22	3.79	5.83	17.4	11.68	10.73	11.1	7.27	5.35
Bdellovibrio	***Microbacterium***	***P3OB.42***	***Holophaga***	***Sorangium***	***Chryseolinea***	***Tahibacter***	***Comamonadaceae***	***Streptomyces***
5.86	5.67	14.4	12.2	14.7	8.83	10.16	4.75	4.60
**Family (alpha=0.05)**								
Sphingobacteriaceae	***Paenibacillaceae***	***Microscillaceae***	***Opitutaceae***	***Caulobacteraceae***	***Comamonadaceae***	***Chitinophagaceae***	***Alcaligenaceae***	**Nocardiaceae**
6.43	4.22	8.30	5.29	7.27	9.90	5.06	9.07	5.23
Micromonosporaceae	***Holophagaceae***	***Labraceae***	***Polyangiaceae***	***Rhodanobacteraceae***	***Weeksellaceae***	***Streptomycetaceae***	***Solirubrobacteraceae***	
4.54	14.1	9.20	13.1	13.4	5.09	4.60	6.26	

Values calculated with 1,000 permutations. Source data are provided as a Source Data file.

Supplementary Table 3. Confidence intervals and percentage of variance (R^2) for significant associations between bacterial ASVs and barley loci.

Taxa (ASV)	***Parental line***	***Chr.***	***Lower marker***	***Upper marker***	***R^2 within the interval per taxa***
Vicinamibacterales	wild	3H	SCRI_RS_130264 (8.8 cM)	SCRI_RS_229894 (20.4 cM)	14.13 %
Variovorax	wild	3H	SCRI_RS_154747 (38.75 cM)	BOPA2_12_10114 (39.4 cM)	63.33 %
Holophaga	wild	3H	SCRI_RS_154747 (38.75 cM)	SCRI_RS_141171 (40.6 cM)	79.47 %
Sorangium	wild	3H	SCRI_RS_154747 (38.75 cM)	No marker at determined genetic position (40 cM)	25.44 %
Tahibacter	wild	3H	SCRI_RS_154747 (38.75 cM)	SCRI_RS_141171 (40.6 cM)	88.03 %
Streptomyces4	elite	4H	No marker at determined genetic position (72.5 cM)	No marker at determined genetic position (77.5 cM)	37.81 %
Stenotrophomonas	wild	4H	SCRI_RS_188944 (94.1 cM)	No marker at determined genetic position (95 cM)	18.46 %
Vicinamibacterales	wild	5H	SCRI_RS_206565 (96.6 cM)	No marker determined	13.83 %

Alpha = 0.05. Source data are provided as a Source Data file

Supplementary Table 4. Confidence intervals and percentage of variance (R^2) for significant associations between bacterial genera and barley loci.

Taxa (Genus)	*Parental line*	*Chr.*	*Lower marker*	*Upper marker*	*R^2 within the interval per taxa*
Ramlibacter	wild	2H	No marker at determined genetic position (117.5 cM)	BOPA2_12_10579	39.47 %
Variovorax	wild	3H	BOPA1_2765_406 (38 cM)	SCRI_RS_141171 (40.6 cM)	60.60 %
Rhodanobacter	wild	3H	No marker at determined genetic position (30 cM)	SCRI_RS_154747 (38.75 cM)	72.47 %
Holophaga	wild	3H	SCRI_RS_154747 (38.75 cM)	SCRI_RS_141171 (40.6 cM)	76.70 %
Sorangium	wild	3H	SCRI_RS_154747 (38.75 cM)	No marker at determined genetic position (40 cM)	30.65 %
Tahibacter	wild	3H	SCRI_RS_154747 (38.75 cM)	SCRI_RS_141171 (40.6 cM)	79.51 %
Microbacterium	wild	5H	SCRI_RS_237352 (95.5 cM)	BOPA2_12_30867 (126.15 cM)	35.29 %
Streptomyces	elite	7H	BOPA1_5595_297 (133.9 cM)	SCRI_RS_6252 (140.1 cM)	32.04 %

Alpha = 0.05. Source data are provided as a Source Data file

Supplementary Table 5. Confidence intervals and percentage of variance (R^2) for significant associations between bacterial families and barley loci.

Taxa (Family)	***Parental line***	***Chr.***	***Lower marker***	***Upper marker***	***R^2 within the interval per taxa***
Polyangiaceae (Sorangium)	wild	1H	SCRI_RS_236160 (97.9 cM)	SCRI_RS_236160 (97.9 cM)	7.93 %
Comamonadaceae (Variovorax)	wild	3H	SCRI_RS_154747 (38.75 cM)	SCRI_RS_141171 (40.6 cM)	67.10 %
Polyangiaceae (Sorangium)	wild	3H	SCRI_RS_154747 (38.75 cM)	No marker at determined genetic position (40 cM)	19.59 %
Holophagaceae (Holophaga)	wild	3H	No marker at determined genetic position (30 cM)	SCRI_RS_141171 (40.6 cM)	74.08 %
Rhodanobacteraceae (*Rhodanobacter* and *Tahibacter)*	wild	3H	SCRI_RS_154747 (38.75 cM)	SCRI_RS_141171 (40.6 cM)	94.26 %
Sphingobacteriaceae	wild	5H	No marker at determined genetic position (155 cM)	SCRI_RS_167103 (161.7 cM)	42.31 %
Streptomycetaceae	elite	7H	BOPA1_5595_297 (133.9 cM)	SCRI_RS_6252 (140.7 cM)	31.13 %

Alpha = 0.05. Source data are provided as a Source Data file

Supplementary Table 6. Marker composition and pedigree analysis of plant genotypes described in this study.

Genotype	Markers number	Heterozygous markers (%)	Maker identity with Barke (%)	Marker identity with a Barke x HID144 Derived (%)
Barke	43,364	0.8	100	n/a
124_52	43,057	1.5	93.3	99.3
124_17	43,302	0.8	95.5	99.9
HID144	42,833	0.9	60.3	n/a

Data based on at least two independent lines per genotype processed with the 50k Illumina Infinium iSelect genotyping platform and calculated using the Flapjack software. *Source data are provided as a Source Data file.*

Supplementary Table 7. Root macro architectural traits of the sibling and the elite lines.

Genotype	*Dry shoot weight (g)*	*Dry root weight (g)*	*Primary root length (cm)*	*Surface area (cm^2)*	*Average diameter (mm)*	*Root Volume (cm^3)*	*Specific root length*	*Root density (g/cm^3)*	*Tips*	*Forks*	*Crosslinks*
**Elite**	0.32 ±0.05	0.29 ±0.17	48 ±5	291 ±7	2.2 ±0.4	18 ±6	2804 ±0.01	0.022 ±0.009	1475 ±3	4328 ±9	129 ±71
**124_17**	0.38 ±0.05	0.34 ±0.19	44 ±6	387 ±35	3.0 ±0.4	30 ±6	2212 ±676	0.018 ±0.013	1481 ±2	4874 ±6	113 ±26
**124_52**	0.37 ±0.03	0.13 ±0.01	41 ±2	312 ±31	2.1 ±0.3	17 ±4	3831 ±348	0.009 ±0.002	1204 ±2	4011 ±8	105 ±27

The values represent the average values (n=4) the standard error of the mean (SEM). Differences were tested using a Kruskal-Wallis test or ANOVA, p-value<0.05. Source data are provided as a Source Data file.

Supplementary Table 8. RNA-seq metadata table. Brep stand for biological replicate, while srep is sequencing replicate.

GENOTYPE	BREP	SREP	SAMPLING DATE	*QRMC-3HS* LOCUS GENO TYPE	QUANT FILES
**124_17**	brep1	srep1	08/05/2019	Elite	CEM01_JH15-105
**124_17**	brep2	srep1	06/05/2019	Elite	CEM01_JH15-14
**124_17**	brep3	srep1	06/05/2019	Elite	CEM01_JH15-26
**124_17**	brep4	srep1	06/05/2019	Elite	CEM01_JH15-51
**124_17**	brep5	srep1	06/05/2019	Elite	CEM01_JH15-7
**124_52**	brep1	srep1	08/05/2019	Wild	CEM01_JH15-104
**124_52**	brep2	srep1	08/05/2019	Wild	CEM01_JH15-115
**124_52**	brep3	srep1	06/05/2019	Wild	CEM01_JH15-32
**124_52**	brep4	srep1	06/05/2019	Wild	CEM01_JH15-35
**124_52**	brep5	srep1	07/05/2019	Wild	CEM01_JH15-95
**BARKE**	brep1	srep1	08/05/2019	Elite	CEM01_JH15-113
**BARKE**	brep2	srep1	08/05/2019	Elite	CEM01_JH15-117
**BARKE**	brep3	srep1	06/05/2019	Elite	CEM01_JH15-22
**BARKE**	brep4	srep1	07/05/2019	Elite	CEM01_JH15-91
**BARKE**	brep5	srep1	07/05/2019	Elite	CEM01_JH15-96

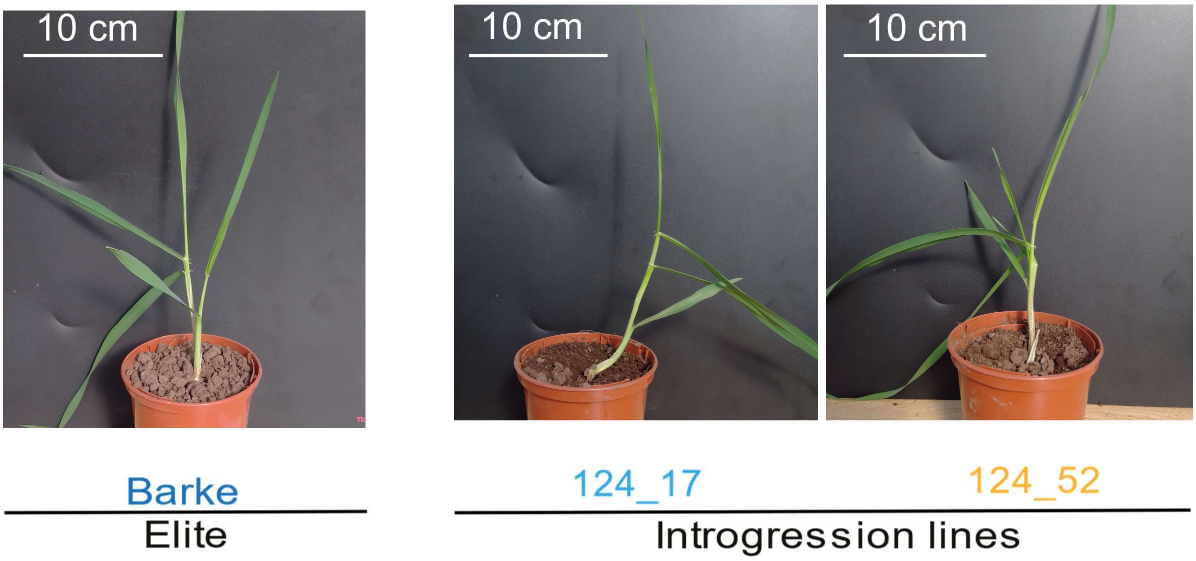

Supplementary Fig. 1. Pictures of the sibling lines 124_52, 124_17 and the elite genotype Barke at elongation stem stage when their rhizosphere microbiota is harvested.

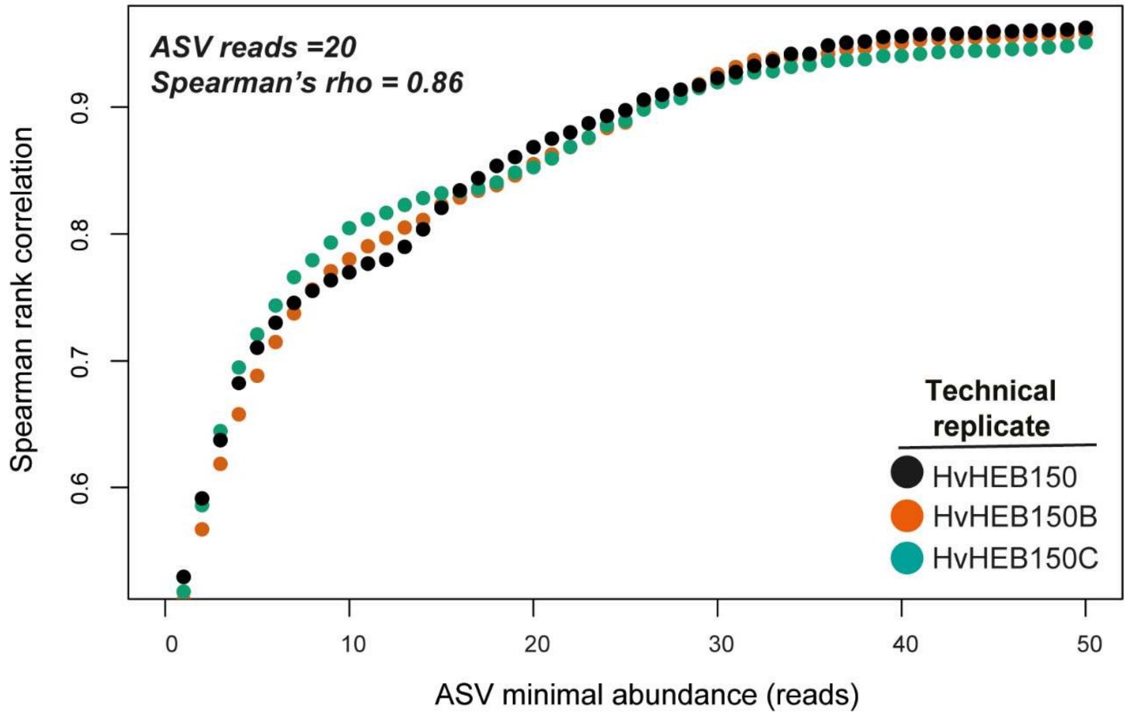

Supplementary Fig. 2. Technical replicate pairwise correlations to assess technical reproducibility at minimum sample size for downstream reads filtering. Source data are provided as a Source Data file.

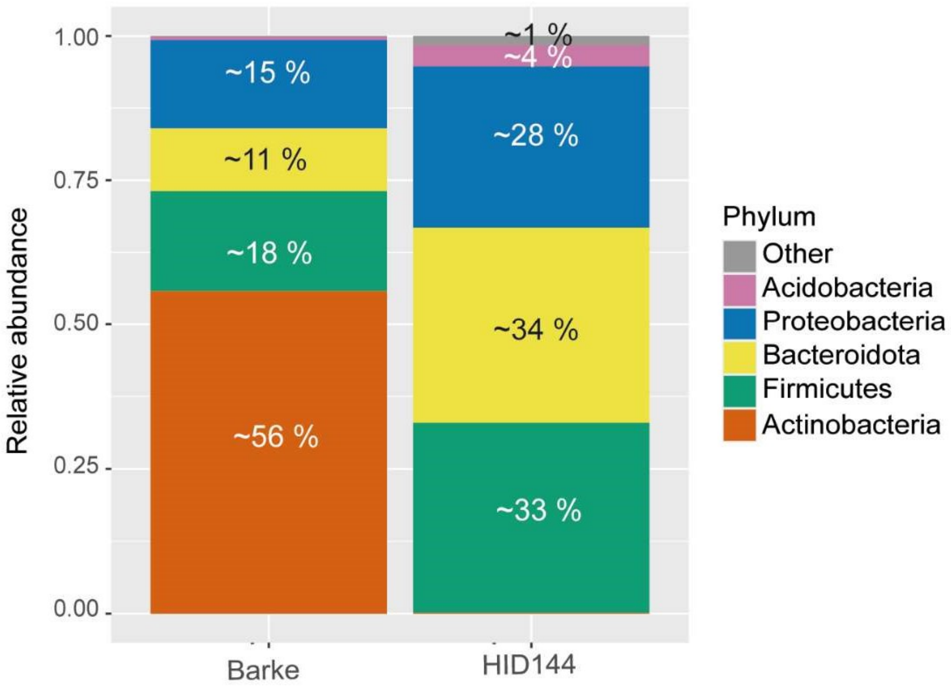

Supplementary Fig. 3. Stacked bar plots showing the main phyla differentially abundant between the parental lines Barke (elite) and HID144 (wild). Source data are provided as a Source Data file.

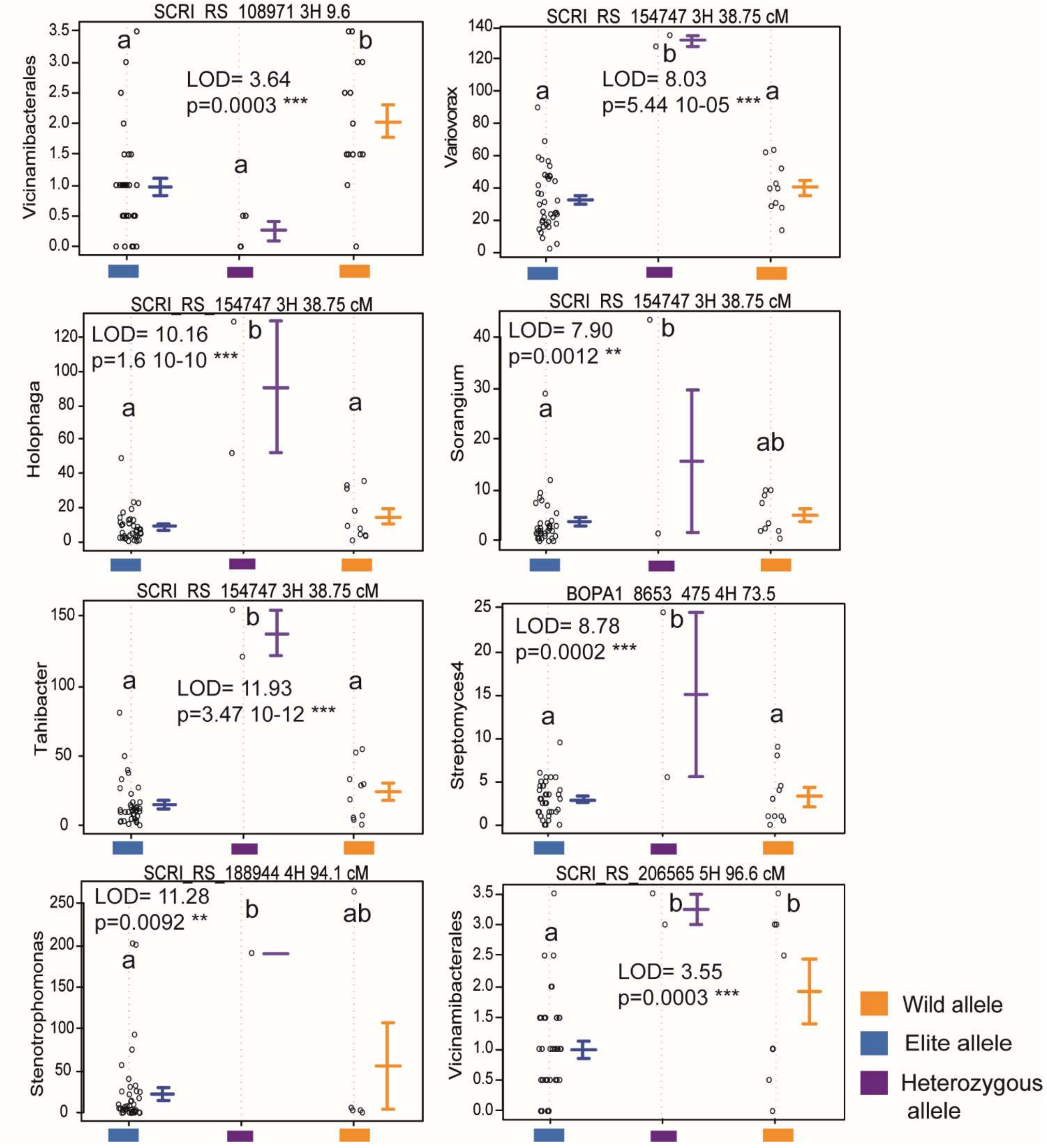

Supplementary Fig. 4. Bacterial abundances in sequencing reads (ASV level, y-axis) in members of the population. Individual dots depict individual biological replicates colour-coded according to allelic composition at molecular markers indicated at the top of each panel with chromosomal location (e.g., 3H, chromosome 3H) and map position in centimorgan (cM). Coloured bars indicate the phenotypic mean values with +/- the standard error. Different lowercase letters denote significant differences calculated using ANOVA post-hoc Tukey or Kruskal–Wallis and post-hoc Dunn's test, at the indicated *P*-values. LOD, Log_{10} likelihood ratio of a QTL presence at that marker position. Source data are provided as a Source Data file.

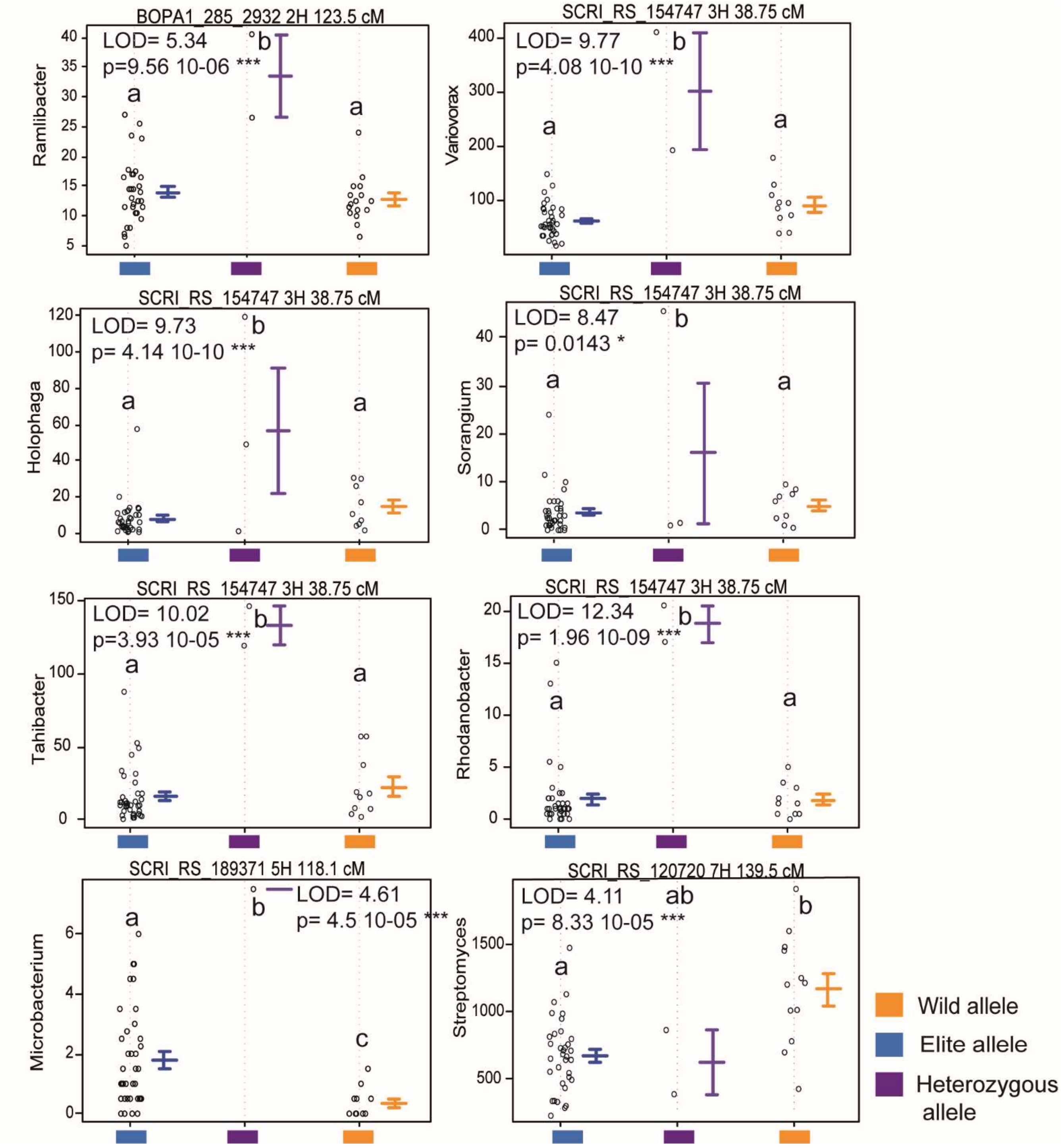

Supplementary Fig. 5. Bacterial abundances in sequencing reads (genus level, y-axis) in members of the population. Individual dots depict individual biological replicates colour-coded according to allelic composition at molecular markers indicated at the top of each panel with chromosomal location (e.g., 3H, chromosome 3H) and map position in centimorgan (cM). Coloured bars indicate the phenotypic mean values with +/- the standard error. Different lowercase letters denote significant differences calculated using ANOVA post-hoc Tukey or Kruskal–Wallis and post-hoc Dunn's test, at the indicated *P*-values. LOD, Log_{10} likelihood ratio of a QTL presence at that marker position. Source data are provided as a Source Data file.

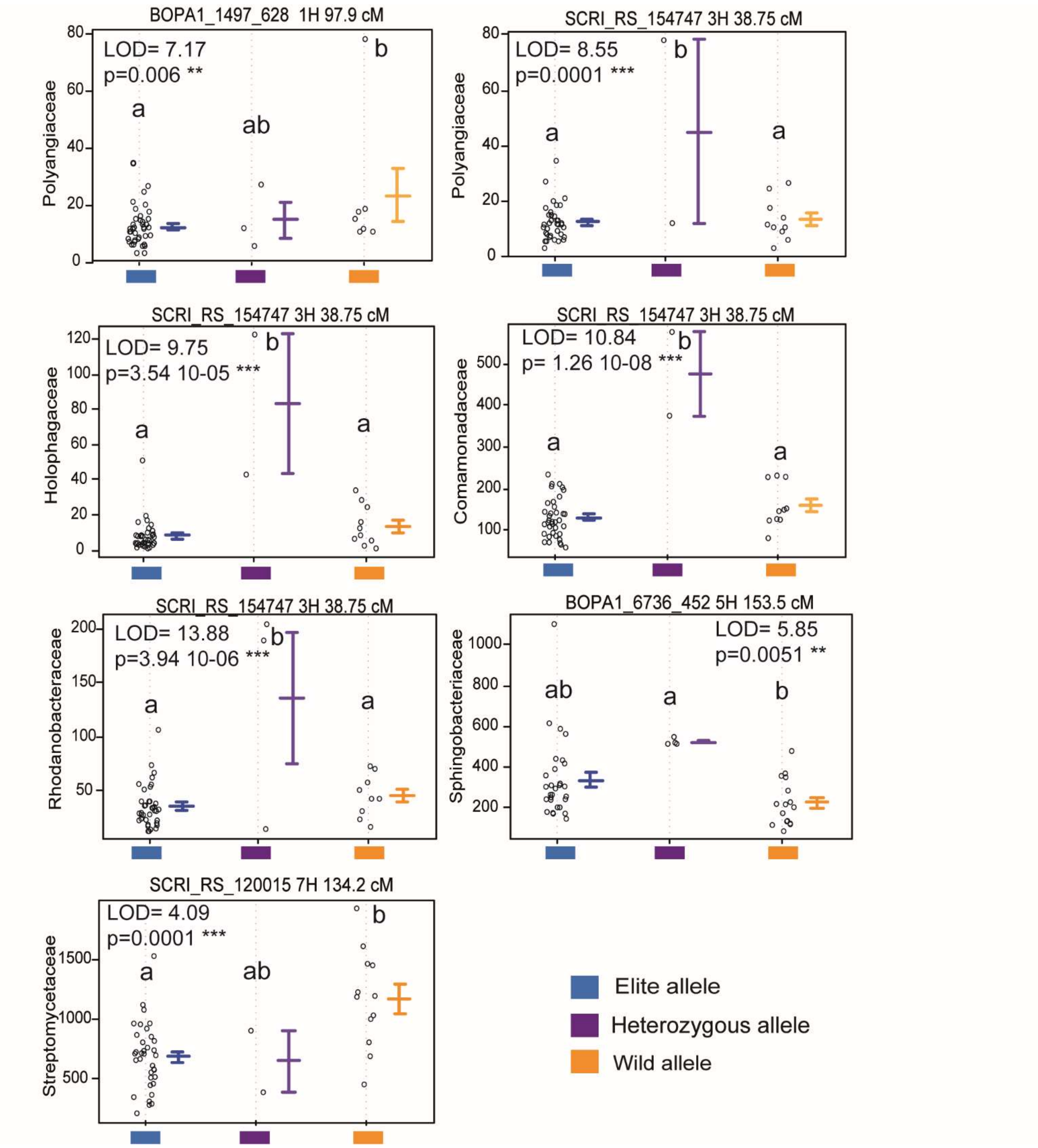

Supplementary Fig. 6. Bacterial abundances in sequencing reads (family level, y-axis) in members of the population. Individual dots depict individual biological replicates colour-coded according to allelic composition at molecular markers indicated at the top of each panel with chromosomal location (e.g., 3H, chromosome 3H) and map position in centimorgan (cM). Coloured bars indicate the phenotypic mean values with +/- the standard error. Different lowercase letters denote significant differences calculated using ANOVA post-hoc Tukey or Kruskal–Wallis and post-hoc Dunn's test, at the indicated *P*-values. LOD, Log_{10} likelihood ratio of a QTL presence at that marker position. Source data are provided as a Source Data file.

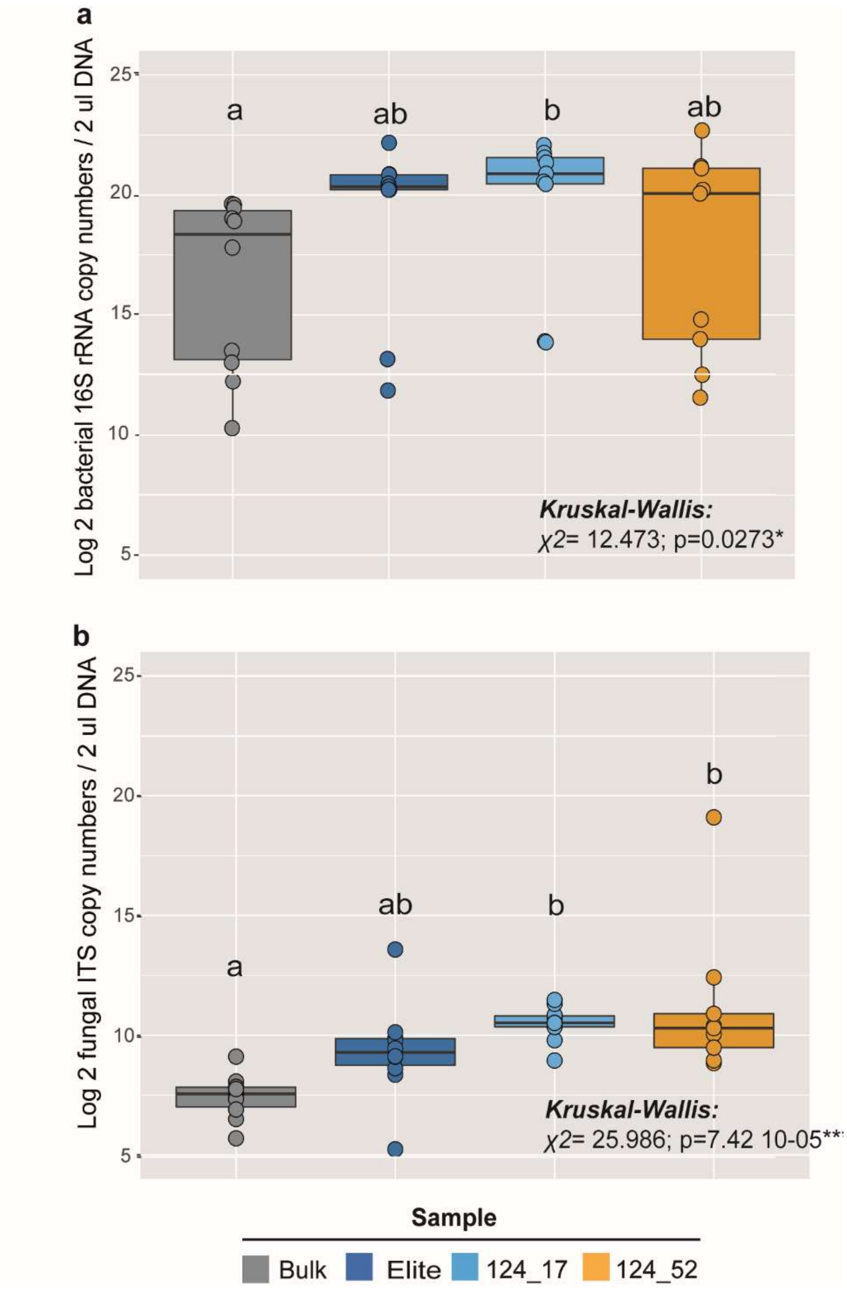

Supplementary Fig. 7. Quantification of bacterial and fungal DNA in the sibling lines, the elite genotype and bulk soil. a) Boxplots depicting the logarithm (base 2) of the number of 16S copies per ul of sample DNA. **b)** Boxplots illustrating the logarithm (base 2) of the number of ITS copies per ul of sample DNA. In each panel, individual dots depict individual biological replicates. Upper and lower edges of the box plots represent the upper and lower quartiles, respectively. The bold line within the box denotes the median. Whiskers denote values within 1.5 interquartile ranges. Different letters indicate significantly different samples (Kruskal–Wallis and post-hoc Dunn's test, at the indicate P values). Source data are provided as a Source Data file.

.

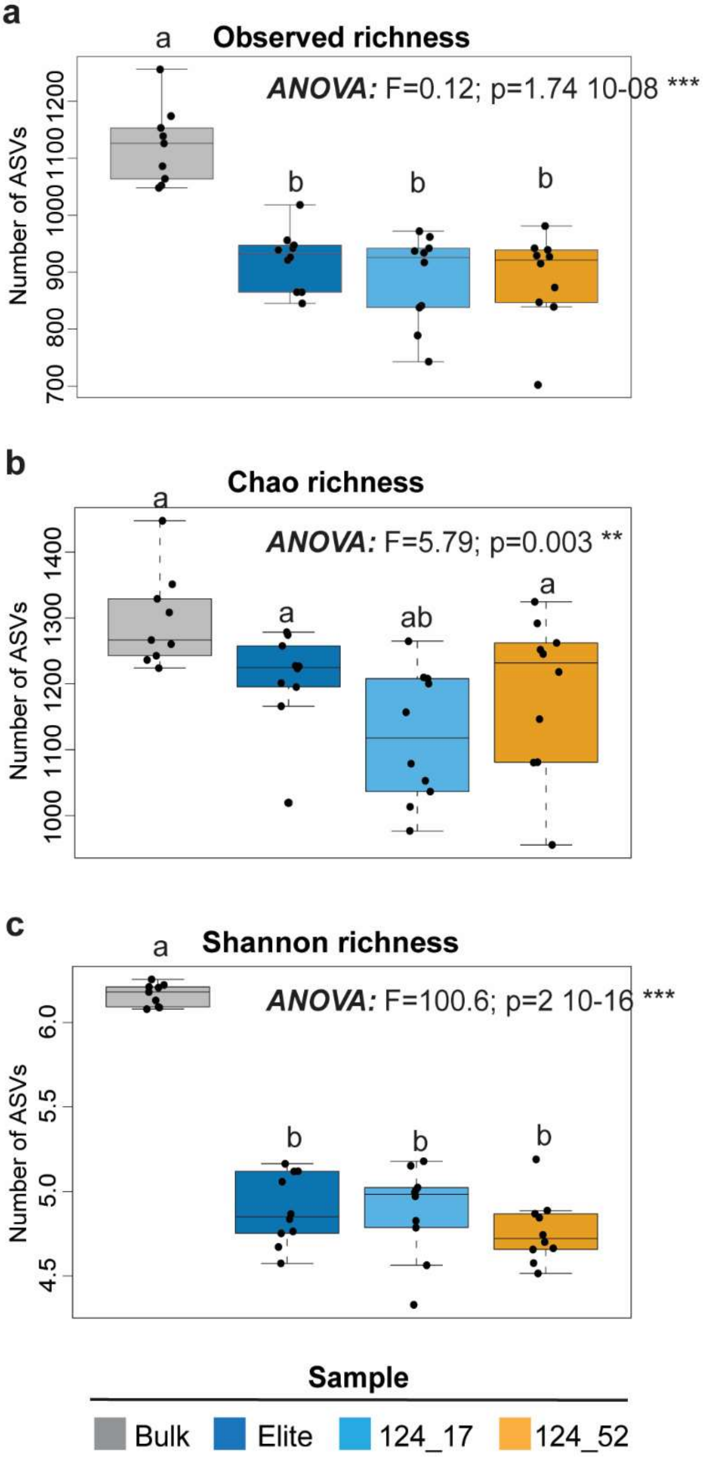

Supplementary Fig. 8. Boxplots depicting alpha-diversity indexes. a) and **b)** show the community richness (observed species, Chao1) and **c)** diversity (Shannon) for the unplanted soil, elite and the sibling lines genotypes. In each panel, individual dots depict individual biological replicates. Upper and lower edges of the box plots represent the upper and lower quartiles, respectively. The bold line within the box denotes the median. Whiskers denote values within 1.5 interquartile ranges. Letters indicate significant differences following ANOVA and post-hoc Tukey test as indicated in the individual panels. Source data are provided as a Source Data file.

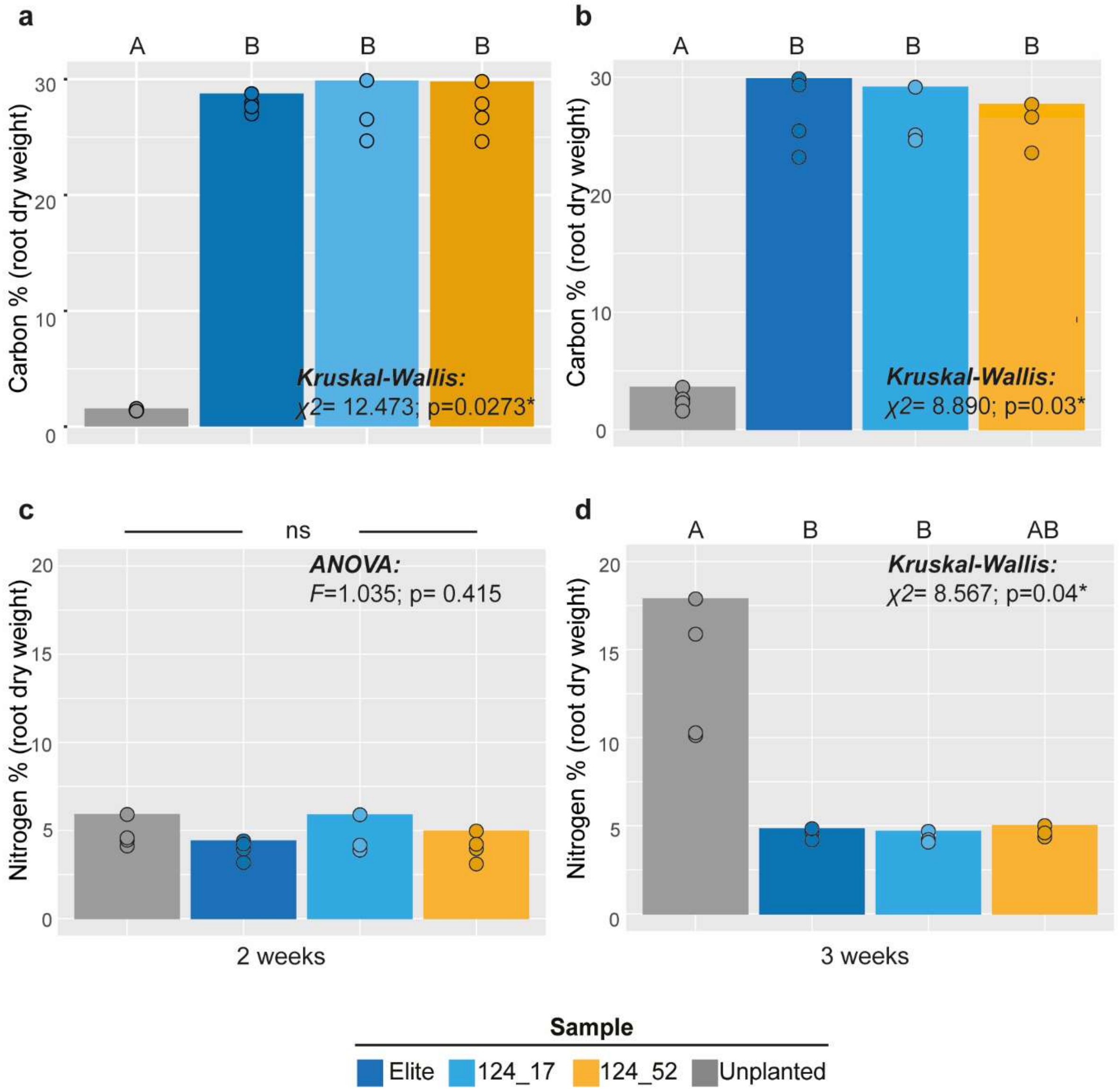

Supplementary Fig. 9. Bar plots representing the mean carbon and nitrogen content (% per weight) in the exudates of the sibling lines, the elite genotypes exudates and the unplanted control at two timepoints. Individual dots depict biological replicates. **a)** Carbon content at 2 weeks timepoint. **b)** Carbon content at 3 weeks timepoint. **c)** Nitrogen content at 2 weeks timepoint. **d)** Nitrogen content at 3 weeks timepoint. In each panel, the upper edge of the box depicts mean value, individual dots are individual biological replicates. Uppercase letters denote significant differences at the indicated statistic; ns no significant differences at the imposed threshold. Source data are provided as a Source Data file.

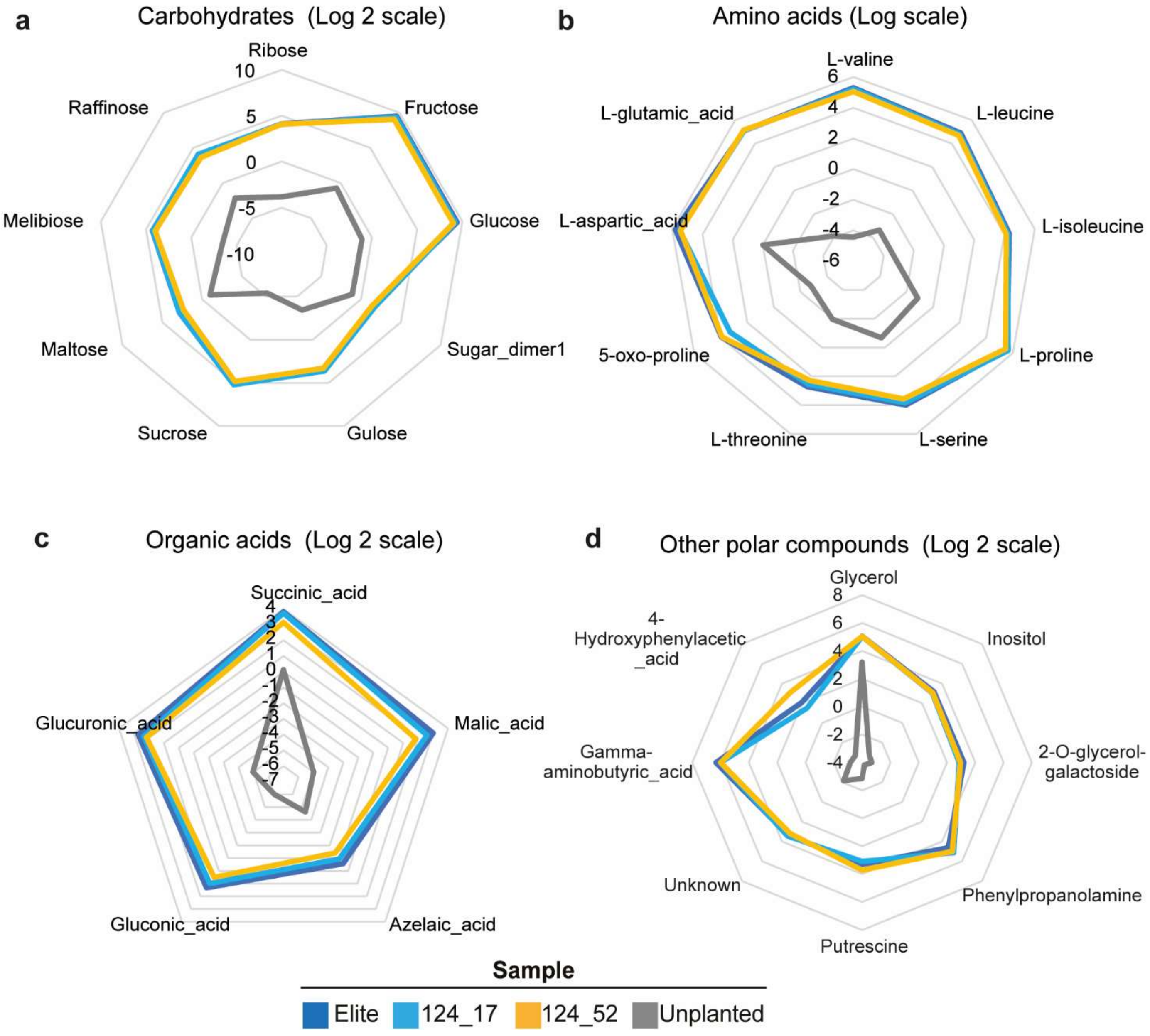

Supplementary Fig. 10. Radar plots depicting the primary metabolism of root exudates and unplanted controls. Different panels indicate categories of compounds retrieved from root exudates **a)** carbohydrates, **b)** amino acids, **c)** organic acids and **d)** other polar compounds. Yellow, blue and light blue lines represent different plant genotypes exudates composition. Grey lines indicate the unplanted control. Individual numbers depict $\log_2$ scaled to the means of four blocks representing cumulative 60 biological replicates per genotype. Source data are provided as a Source Data file.

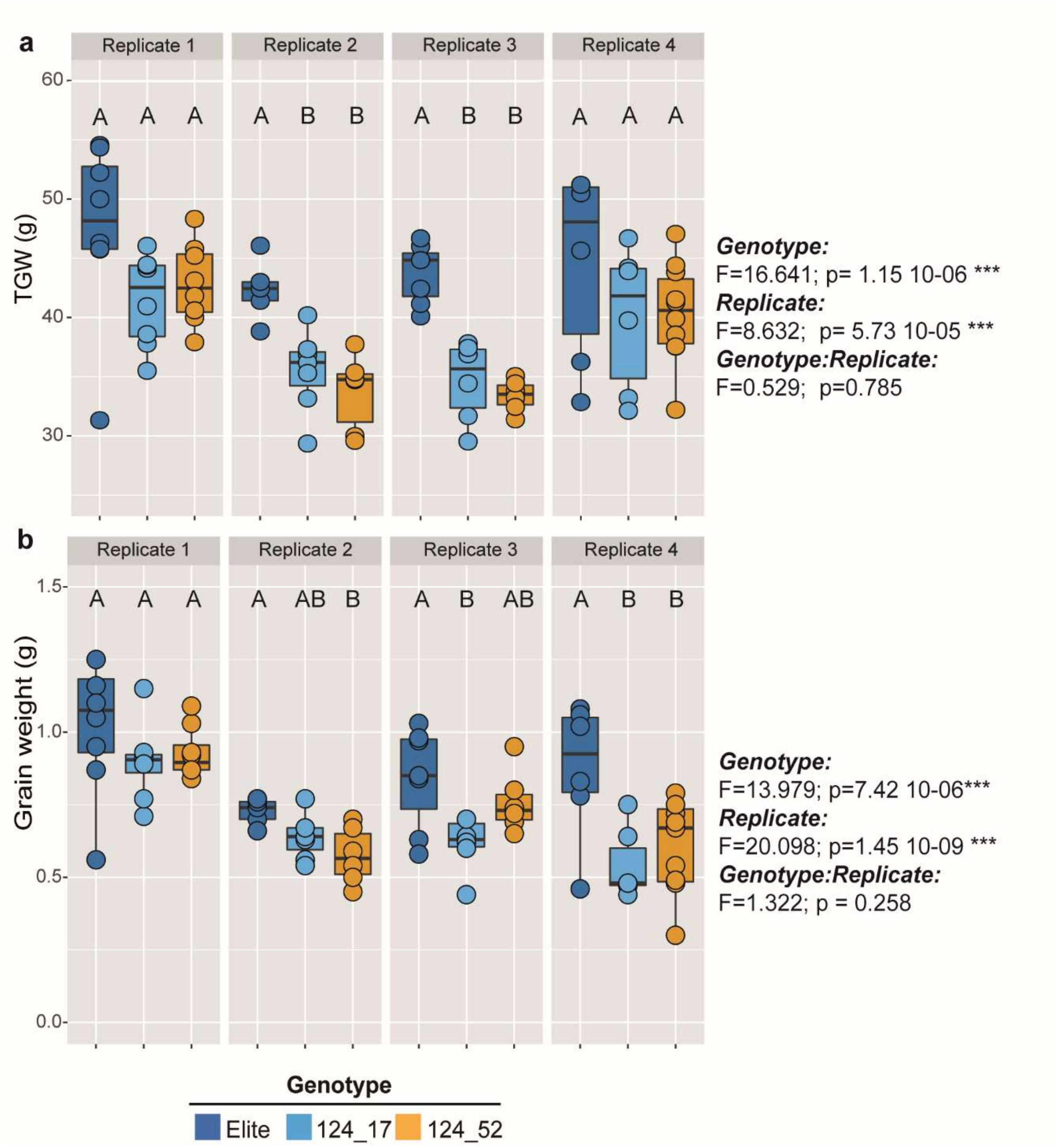

Supplementary Fig. 11. Boxplot representing different barley yield parameters. a) thousand grain weight and **b)** total yield (in grams) per main tiller in the sibling lines and the elite genotype Barke. In each panel, data is shown in four independent experiments to observe the significant effect of the replication. Individual dots depict individual biological replicates. Upper and lower edges of the box plots represent the upper and lower quartiles, respectively. The bold line within the box denotes the median. Whiskers denote values within 1.5 interquartile ranges. Uppercase letters denote significant differences determined using an ANOVA followed by post-hoc Tukey test for the factors as indicated at the side of the panel. Source data are provided as a Source Data file.

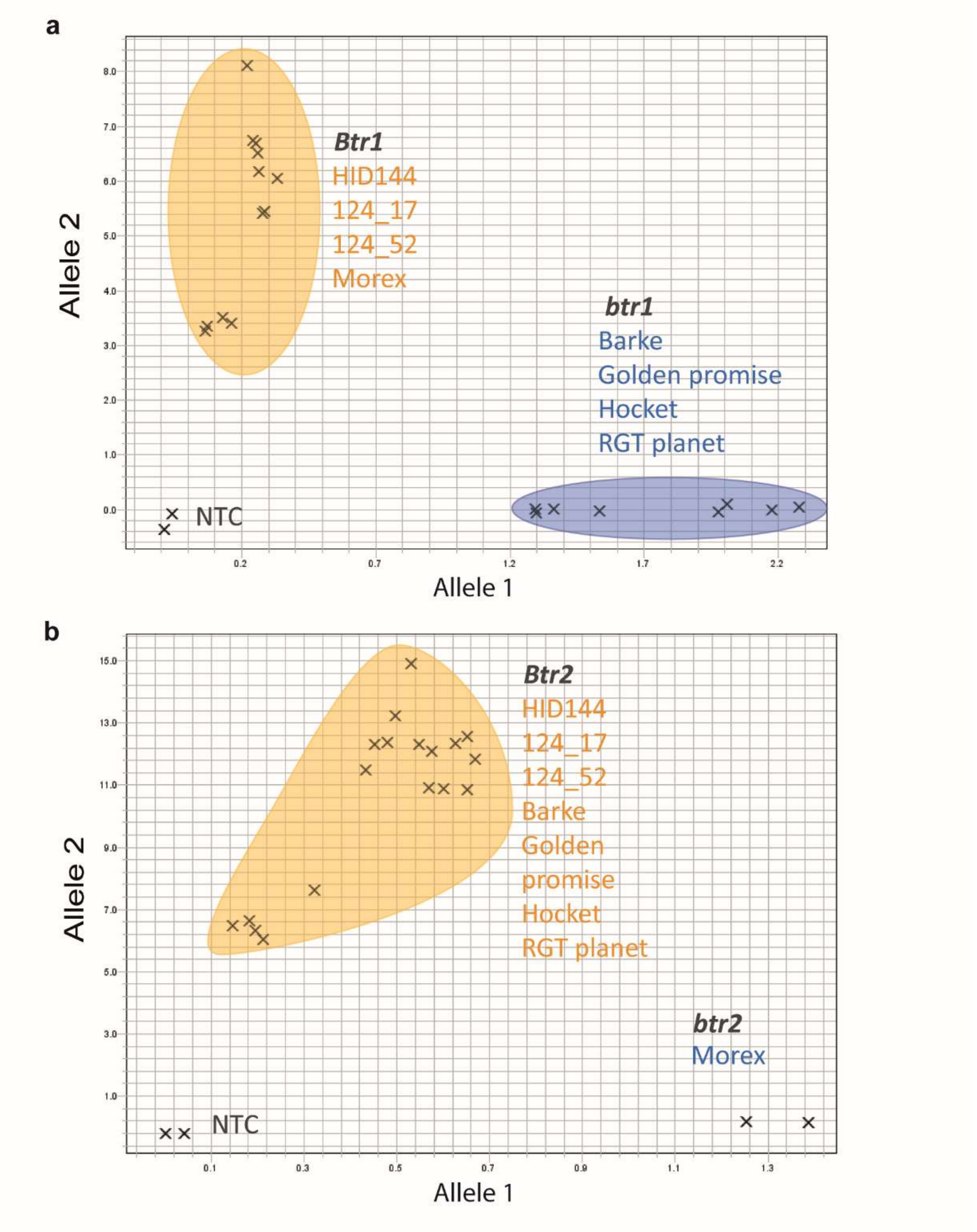

Supplementary Fig. 12. Allele discrimination scatter plot for mutations in the brittle rachis genes a) *Btr1* and b) *Btr2* in the genotypes HID144, Barke, 124_17, 124_52, Morex, Golden Promise, Hockett and RGT planet. The x-axis in **a)** represents the relative fluorescent emission for the C allele-specific probe and the y-axis represents the represents the relative fluorescent emission for the G Allele. The x-axis in **b)** represents the relative fluorescent emission for the T allele-specific probe and the y-axis represents the represents the relative fluorescent emission for the G Allele. NTC represents no template control. Source data are provided as a Source Data file.

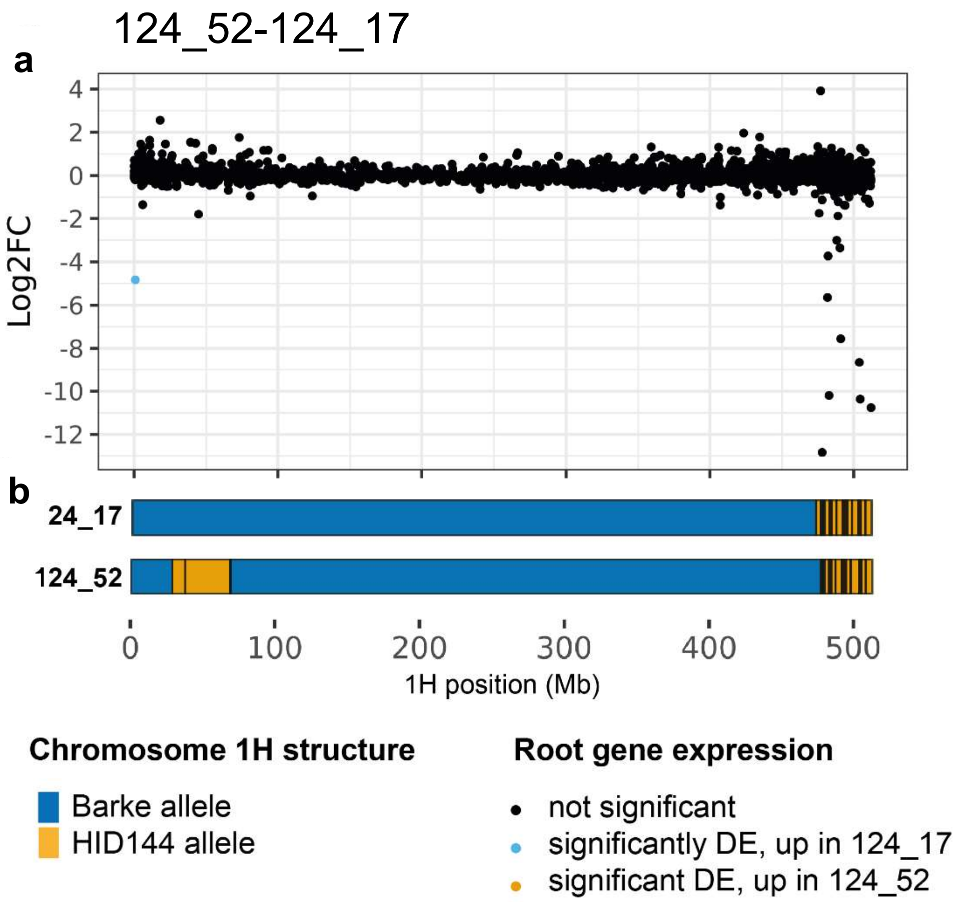

Supplementary Fig. 13. Relationship between chromosomal composition and transcriptional profiles computed for genes mapping on chromosome 1H. a) $\log_2$ fold changes of genes with their position on barley chromosome 1H, compared to **b)** allelic information for lines 124_17 and 124_52. HID-144 introgressions are illustrated in yellow, Barke background is shown in blue. Genes are coloured according to significance, with non-DE genes coloured in black, those genes upregulated in 124_52 when compared to 124_17 in yellow, and those downregulated in the same comparison in blue. Source data are provided as a Source Data file.

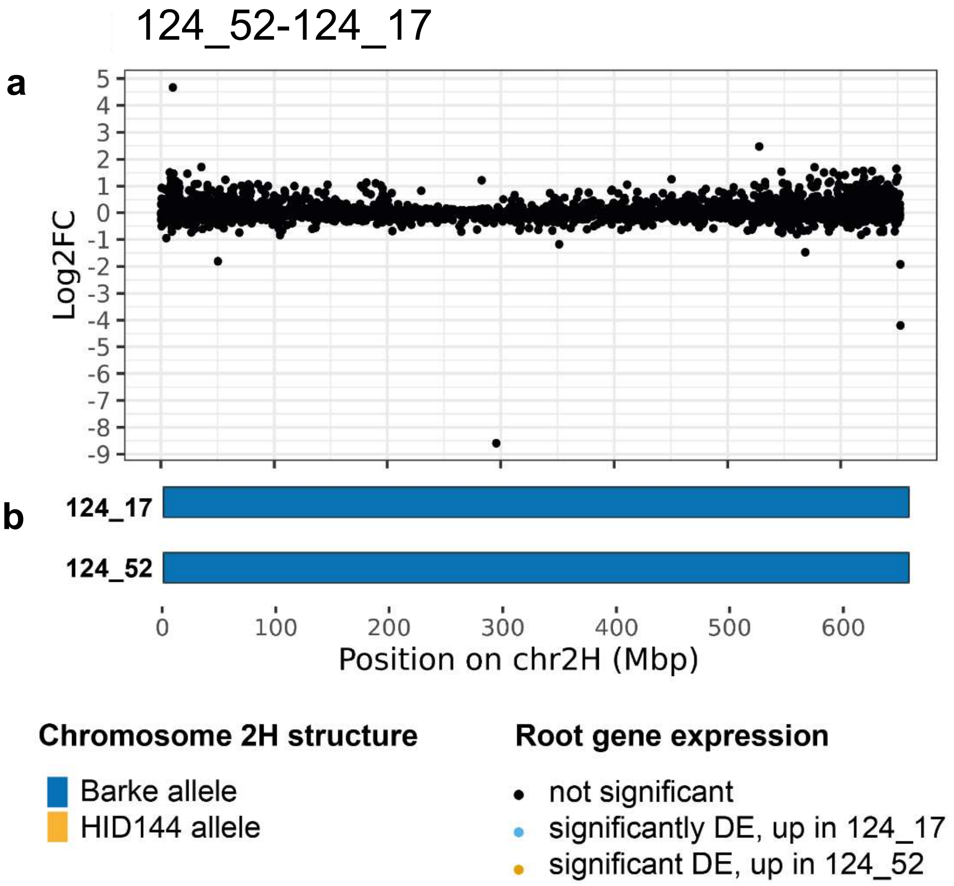

Supplementary Fig. 14. Relationship between chromosomal composition and transcriptional profiles computed for genes mapping on chromosome 2H. a) $\log_2$ fold changes of genes with their position on barley chromosome 2H, compared to **b)** allelic information for lines 124_17 and 124_52. HID-144 introgressions are illustrated in yellow, Barke background is shown in blue. Genes are coloured according to significance, with non-DE genes coloured in black, those genes upregulated in 124_52 when compared to 124_17 in yellow, and those downregulated in the same comparison in blue. Source data are provided as a Source Data file.

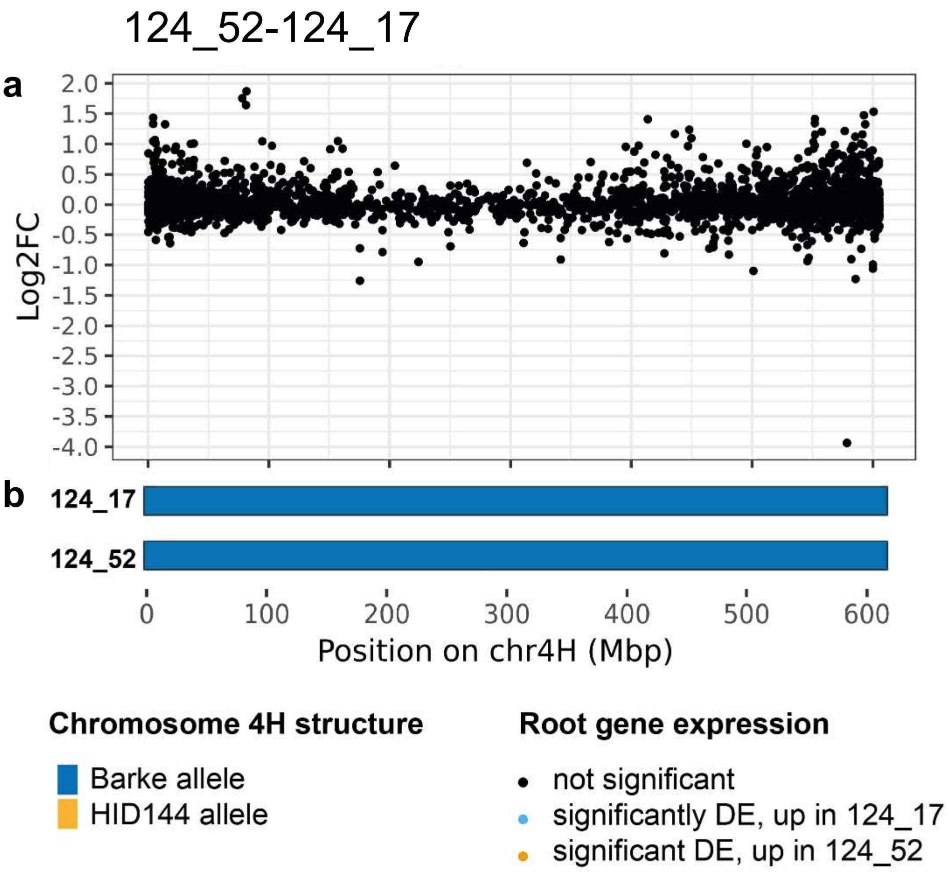

Supplementary Fig. 15. Relationship between chromosomal composition and transcriptional profiles computed for genes mapping on chromosome 4H. a) $\log_2$ fold changes of genes with their position on barley chromosome 4H, compared to **b)** allelic information for lines 124_17 and 124_52. HID-144 introgressions are illustrated in yellow, Barke background is shown in blue. Genes are coloured according to significance, with non-DE genes coloured in black, those genes upregulated in 124_52 when compared to 124_17 in yellow, and those downregulated in the same comparison in blue. Source data are provided as a Source Data file.

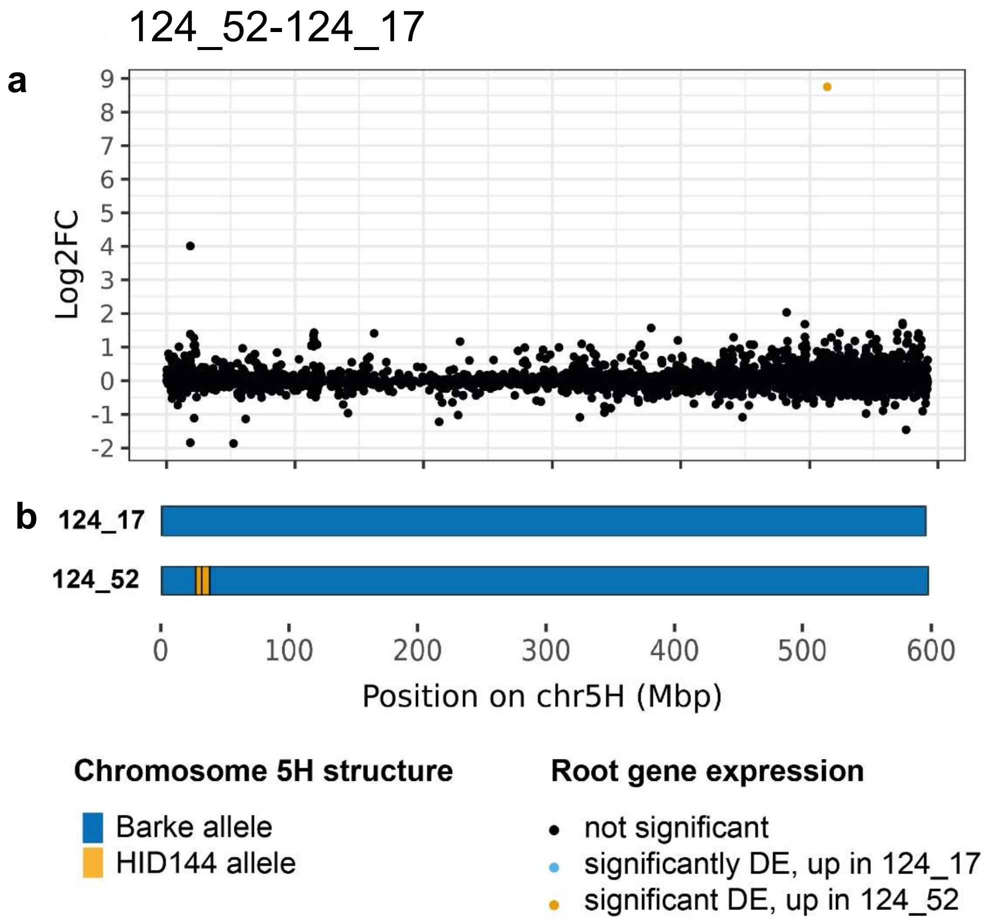

Supplementary Fig. 16. Relationship between chromosomal composition and transcriptional profiles computed for genes mapping on chromosome 5H. a) $\log_2$ fold changes of genes with their position on barley chromosome 5H, compared to **b)** allelic information for lines 124_17 and 124_52. HID-144 introgressions are illustrated in yellow, Barke background is shown in blue. Genes are coloured according to significance, with non-DE genes coloured in black, those genes upregulated in 124_52 when compared to 124_17 in yellow, and those downregulated in the same comparison in blue. Source data are provided as a Source Data file.

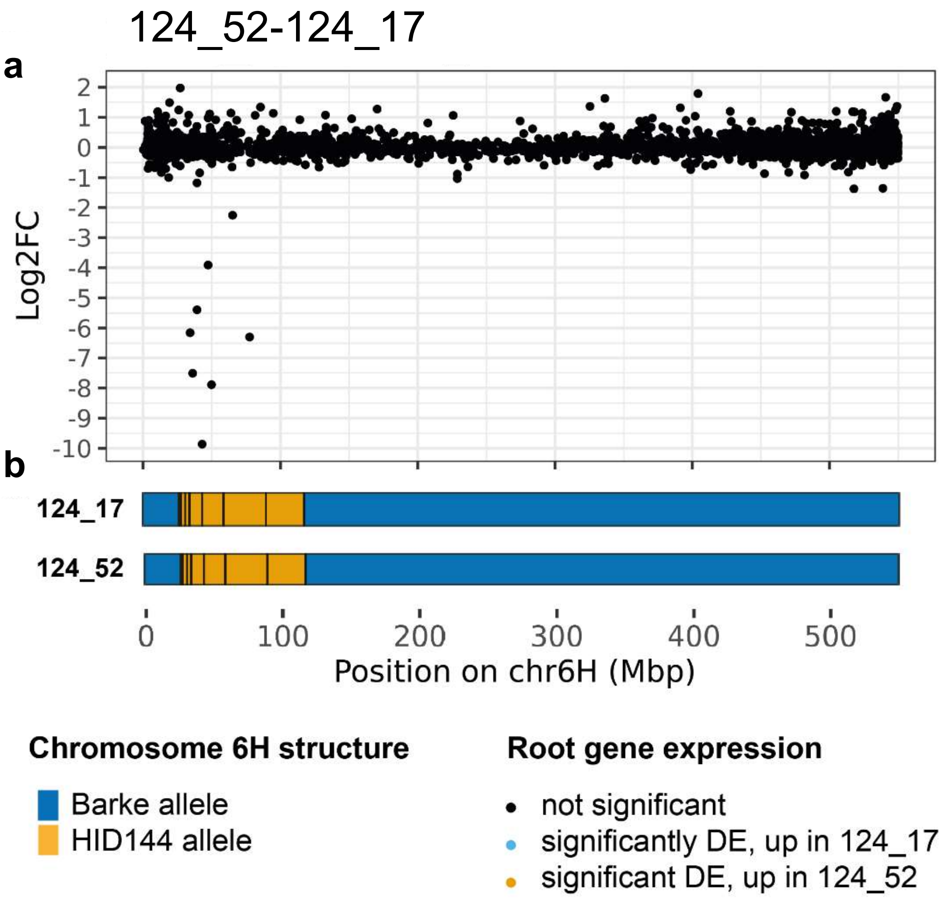

Supplementary Fig. 17. Relationship between chromosomal composition and transcriptional profiles computed for genes mapping on chromosome 6H. a) $\log_2$ fold changes of genes with their position on barley chromosome 6H, compared to **b)** allelic information for lines 124_17 and 124_52. HID-144 introgressions are illustrated in yellow, Barke background is shown in blue. Genes are coloured according to significance, with non-DE genes coloured in black, those genes upregulated in 124_52 when compared to 124_17 in yellow, and those downregulated in the same comparison in blue. Source data are provided as a Source Data file.

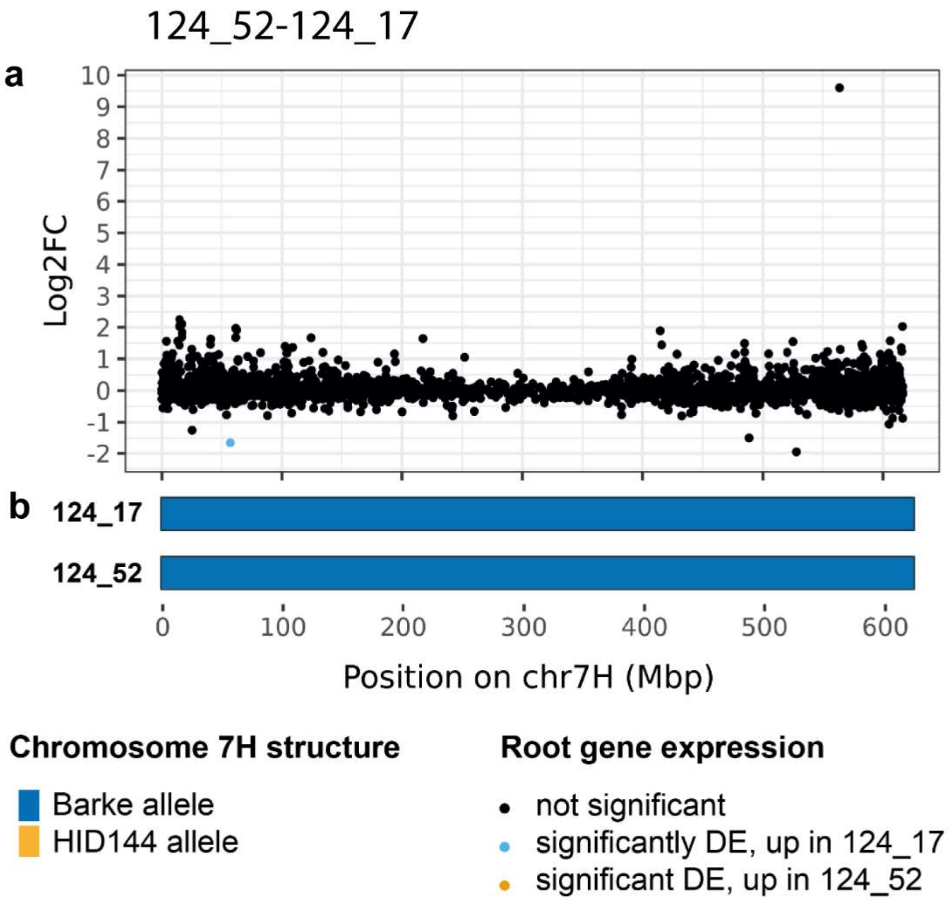

Supplementary Fig. 18. Relationship between chromosomal composition and transcriptional profiles computed for genes mapping on chromosome 7H. a) $\log_2$ fold changes of genes with their position on barley chromosome 7H, compared to **b)** allelic information for lines 124_17 and 124_52. HID-144 introgressions are illustrated in yellow, Barke background is shown in blue. Genes are coloured according to significance, with non-DE genes coloured in black, those genes upregulated in 124_52 when compared to 124_17 in yellow, and those downregulated in the same comparison in blue. Source data are provided as a Source Data file.

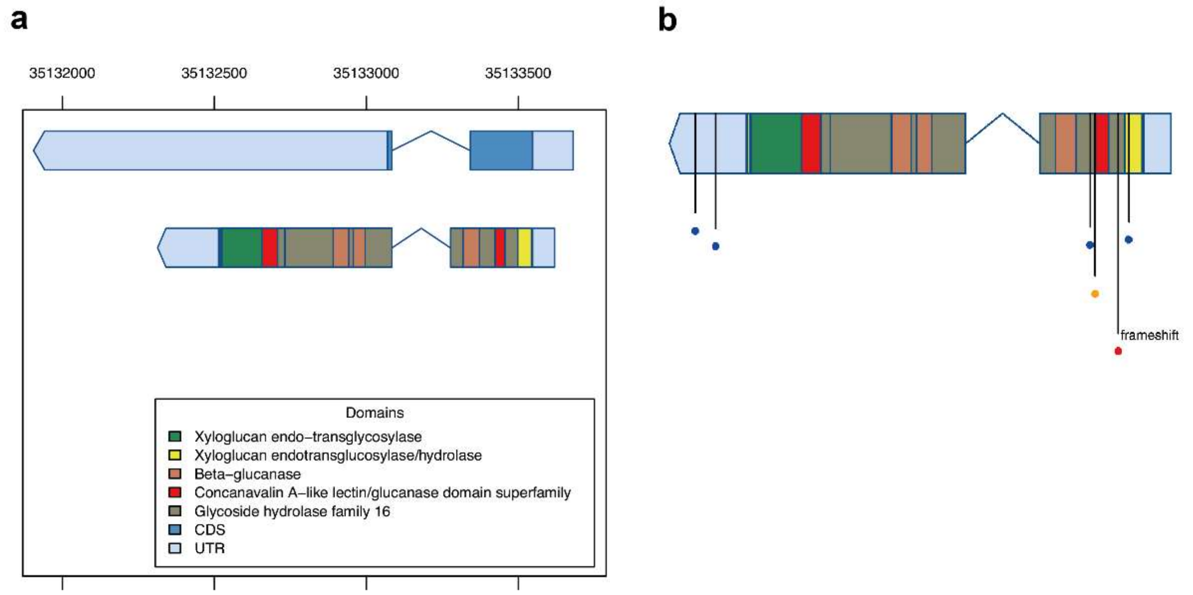

Supplementary Fig. 19. Schematics of transcripts in gene BaRT2v18chr3HG123140 annotated as a xyloglucan endotransglucosylase/hydrolase, illustrating results of variant calling and annotation. a) Two transcripts present in BaRT2v18chr3HG123140, annotated with domains using InterProScan. **b)** The second productive transcript annotated with variants. Low impact variants (those not affecting predicted amino acid sequence) are shown in blue, moderate impact variants (those causing localised amino acid changes) are shown in yellow, and a high impact frameshift variant is shown in red. Source data are provided as a Source Data file.

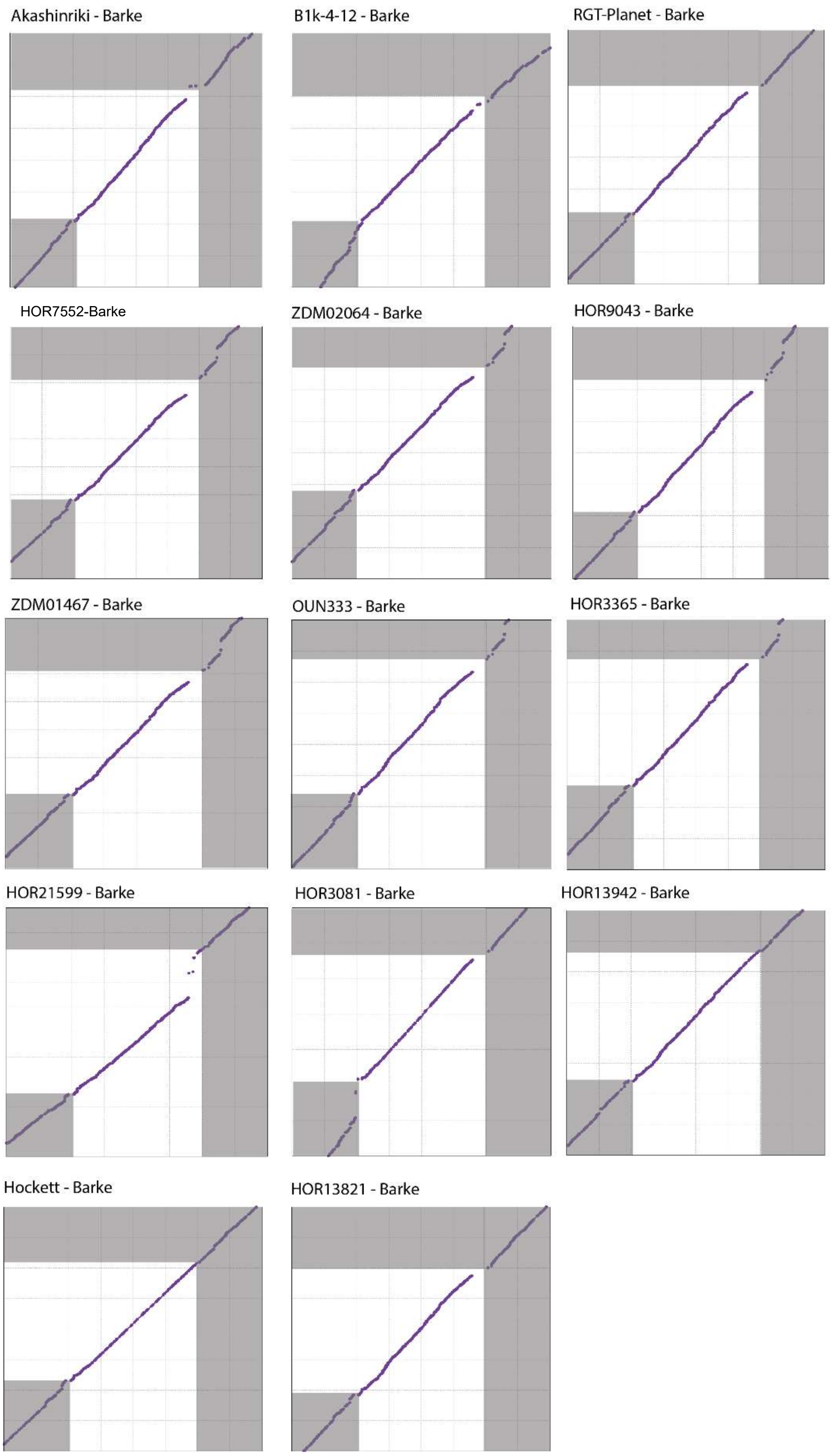

Supplementary Fig. 20. MUMmer plots showing collinearity of sequence between Barke (x-axis) and 14 other pan-genome genotypes (y-axis) in and around the *QRMC-3HS* locus. Area in white shows the position of the *QRMC-3HS* locus, grey areas are outside the *QRMC-3HS*. Purple dots are sequence matches between two genotypes. Source data are provided as a Source Data file.

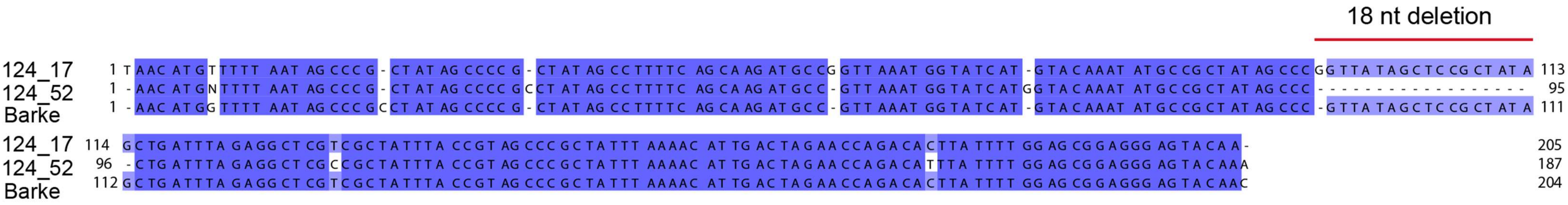

Supplementary Fig. 21. Nucleotide alignment showing the NLR diagnostic marker amplicon sequences (18 nt deletion flanking regions). Sanger sequences of the NLR marker amplicon were used as input. The alignment was generated in Jalview 2.11.2.2 using the Muscle alignment with default parameters. The level of sequence similarity is indicated by dark (high identity) to light purple colour (low identity). Source data are provided as a Source Data file.
