# Supplementary material for: Identifying plant genes shaping microbiota composition in the barley rhizosphere
Source: Nat Commun. 2022 Jun 16;13:3443. doi: 10.1038/s41467-022-31022-y (PMC9203816; doi:10.1038/s41467-022-31022-y)
Supplement: Supplementary file 3 — Description of Additional Supplementary Files [file 41467_2022_31022_MOESM3_ESM.pdf]

Description of Additional Supplementary Files

File Name: Supplementary Data 1
Description: Database containing the LOD score results of the mapping analysis at ASV, genus and family level. Source data are provided as a Source Data file.

File Name: Supplementary Data 2
Description: Database including the statistical comparisons of the polar and non-polar data compounds across different barley genotypes and unplanted control. Source data are provided as a Source Data file.

File Name: Supplementary Data 3
Description: Database describing the 50K iSelect SNP genotyping information of the sibling and the parental lines.

File Name: Supplementary Data 4
Description: Database describing the 34 differentially expressed genes in 124_52 -124_17 comparison group, with information on Log2 FC, physical position, predicted annotation, and underlying genetic information for 124_52 and 124_17. Source data are provided as a Source Data file.

File Name: Supplementary Data 5
Description: : Database describing the 59 BaRTv2 genes with physical positions in the QRMC-3HS locus, with annotation and expression information. Source data are accessible at https://ics.hutton.ac.uk/barleyrtd/bart_v2_18.html.

File Name: Supplementary Data 6
Description: Database containing a description of the results of variant calling for expressed genes with SnpEff annotation. Source data are provided as a Source Data file.
