# Supplementary material for: Identifying plant genes shaping microbiota composition in the barley rhizosphere
Source: Nat Commun. 2022 Jun 16;13:3443. doi: 10.1038/s41467-022-31022-y (PMC9203816; doi:10.1038/s41467-022-31022-y)
Supplement: Supplementary file 10 — Reporting Summary [file 41467_2022_31022_MOESM10_ESM.pdf]

nature portfolio

Corresponding author(s): Davide Bulgarelli

Last updated by author(s): May 27, 2022

Reporting Summary

Nature Portfolio wishes to improve the reproducibility of the work that we publish. This form provides structure for consistency and transparency in reporting. For further information on Nature Portfolio policies, see our Editorial Policies and the Editorial Policy Checklist.

Statistics

For all statistical analyses, confirm that the following items are present in the figure legend, table legend, main text, or Methods section.

n/a	Confirmed	
☐	☒	The exact sample size (*n*) for each experimental group/condition, given as a discrete number and unit of measurement
☐	☒	A statement on whether measurements were taken from distinct samples or whether the same sample was measured repeatedly
☐	☒	The statistical test(s) used AND whether they are one- or two-sided<br>*Only common tests should be described solely by name; describe more complex techniques in the Methods section.*
☐	☒	A description of all covariates tested
☐	☒	A description of any assumptions or corrections, such as tests of normality and adjustment for multiple comparisons
☐	☒	A full description of the statistical parameters including central tendency (e.g. means) or other basic estimates (e.g. regression coefficient) AND variation (e.g. standard deviation) or associated estimates of uncertainty (e.g. confidence intervals)
☐	☒	For null hypothesis testing, the test statistic (e.g. *F*, *t*, *r*) with confidence intervals, effect sizes, degrees of freedom and *P* value noted<br>*Give P values as exact values whenever suitable.*
☒	☐	For Bayesian analysis, information on the choice of priors and Markov chain Monte Carlo settings
☒	☐	For hierarchical and complex designs, identification of the appropriate level for tests and full reporting of outcomes
☒	☐	Estimates of effect sizes (e.g. Cohen's *d*, Pearson's *r*), indicating how they were calculated

Our web collection on statistics for biologists contains articles on many of the points above.

Software and code

Policy information about availability of computer code

Data collection	No code was used to collect data.
Data analysis	We created a repository in GitHub where individual packages, versions and scripts to reproduce data analysis and figures of the manuscript are described in details. The repository is accessible at: https://github.com/BulgarelliD-Lab/Microbiota_mapping. For metabolite profiling data acquisition and processing was performed using the Xcalibur software package V. 2.0.7 (Thermo Scientific, Waltham, MA, USA)

For manuscripts utilizing custom algorithms or software that are central to the research but not yet described in published literature, software must be made available to editors and reviewers. We strongly encourage code deposition in a community repository (e.g. GitHub). See the Nature Portfolio guidelines for submitting code & software for further information.

Data

Policy information about availability of data

All manuscripts must include a data availability statement. This statement should provide the following information, where applicable:

- Accession codes, unique identifiers, or web links for publicly available datasets
- A description of any restrictions on data availability
- For clinical datasets or third party data, please ensure that the statement adheres to our policy

The raw sequence data collected in this study s reported in this manuscript have been deposited in the European Nucleotide Archive (ENA), https://www.ebi.ac.uk/ (accession number PRJEB50061). Source data to generate individual figures and computational analysis are provided with this paper. The barley reference transcriptome (v2.18) for the cultivar Barke was obtained from https://ics.hutton.ac.uk/barleyrtd/bart_v2_18.html. Pseudomolecules of individual barley genomes were downloaded from https://webblast.ipk-gatersleben.de/downloads/barley_pangenome/.

Field-specific reporting

Please select the one below that is the best fit for your research. If you are not sure, read the appropriate sections before making your selection.

☒ Life sciences ☐ Behavioural & social sciences ☐ Ecological, evolutionary & environmental sciences

For a reference copy of the document with all sections, see nature.com/documents/nr-reporting-summary-flat.pdf

Life sciences study design

All studies must disclose on these points even when the disclosure is negative.

Sample size	We used previous investigations of the barley microbiota conducted in the same soil type [i.e., PMID: 28694814; PMID: 32737353; PMID: 34900424] to define a minimum number of biological replicates per parental or sibling genotypes as n=4.
Data exclusions	No data were excluded beyond sequencing reads with low quality scores
Replication	Experiments were not replicated more than once due to the cost of materials, library preparation and sequencing, unless stated otherwise in the text. PCR reactions were performed in triplicates using at least two independent master mixes (i.e., 6 reactions in total per sample) for amplicon library preparation and pooled to control for variation in PCR. Likewise, we used technical replicates to define the reproducibility threshold of amplicon sequencing variants. All attempts at replication were successful.
Randomization	We used a randomised design for the mapping experiment and the characterization of the sibling lines, specifically for the amplicon sequencing, transcriptomic, root architecture and yield experiments. Due to the material requirements for the primary metabolite analysis, this latter experiments was organised as a randomised block design
Blinding	For both sequence-based (i.e., amplicon sequencing, mapping and transcriptomic) and phenotypic analyses (e.g., root phenotypes, primary metabolites, yield) blinding was not relevant as quantifications were performed using automated pipelines applied equally to all replicates preventing researchers to influence and/or systematically bias the acquired data.

Reporting for specific materials, systems and methods

We require information from authors about some types of materials, experimental systems and methods used in many studies. Here, indicate whether each material, system or method listed is relevant to your study. If you are not sure if a list item applies to your research, read the appropriate section before selecting a response.

Materials & experimental systems

n/a	Involved in the study
☒	☐ Antibodies
☒	☐ Eukaryotic cell lines
☒	☐ Palaeontology and archaeology
☒	☐ Animals and other organisms
☒	☐ Human research participants
☒	☐ Clinical data
☒	☐ Dual use research of concern

Methods

n/a	Involved in the study
☒	☐ ChIP-seq
☒	☐ Flow cytometry
☒	☐ MRI-based neuroimaging
