# Supplementary figures and images for: Identifying plant genes shaping microbiota composition in the barley rhizosphere
Source: Nat Commun. 2022 Jun 16;13:3443. doi: 10.1038/s41467-022-31022-y (PMC9203816; doi:10.1038/s41467-022-31022-y)

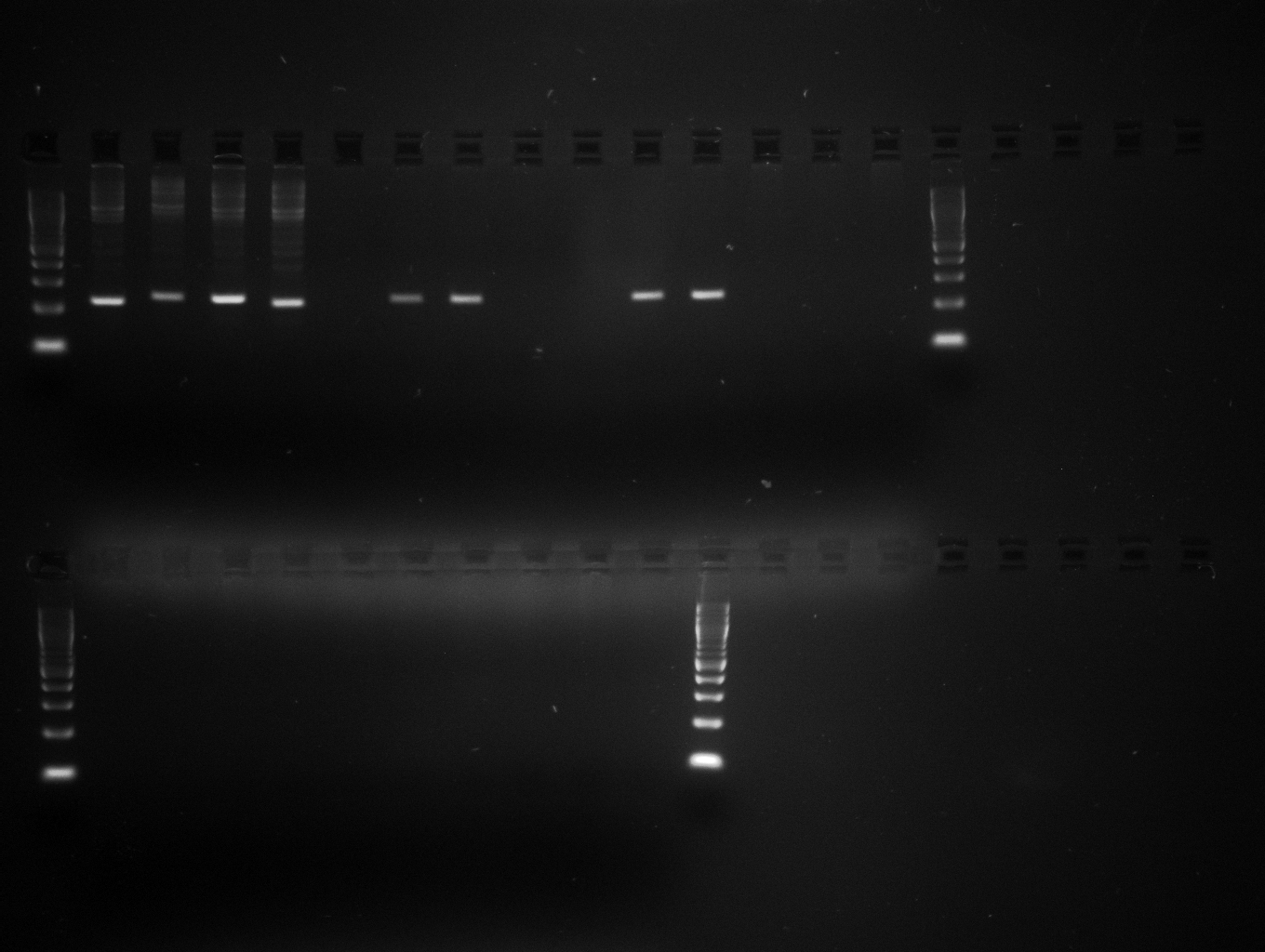

Supplement: Supplementary file 11 — Source Data [file 41467_2022_31022_MOESM11_ESM.zip › Fig 7/QRMC-3HS_Fig7C.Tif]
